# NO/GMP as mediators of estrogen effects in bone

**DOI:** 10.1186/2050-6511-16-S1-A10

**Published:** 2015-09-02

**Authors:** Hema Kalyanaraman, Jisha Joshua, Shunhui Zhuang, Esther Corey, Robert Sah, Renate B Pilz

**Affiliations:** 1 Department of Medicine, University of California, San Diego, La Jolla, California 92093, USA; 2 Department of Bioengineering, University of California, San Diego, La Jolla, California 92093, USA

## Background

Osteoporosis is a major health problem, especially in post-menopausal women. Current osteoporosis therapies have major drawbacks (e.g., estrogens increase breast cancer risks), and reduce fractures by only ~20-30%. Most osteoporosis drugs block bone resorption, and there is an urgent need to develop anabolic therapies that can stimulate bone formation. We previously showed that the NO/cGMP/PKG pathway mediates pro-proliferative effects of mechanical stimulation in osteoblasts and anti-apoptotic effects of estrogens in osteocytes [1,2]. These findings lead us to hypothesize that cGMP-elevating agents may have boneprotective effects.

## Results

We tested cinaciguat, a prototype soluble guanylate cyclase stimulator, and nitrosylcobinamide (NO-Cbi), a novel NO-donor with anti-oxidant properties, in a mouse model of estrogen deficiency-induced osteoporosis [3]. Compared with sham-operated mice, ovariectomized mice had lower serum cGMP concentrations, which were largely restored to normal by treatment with cinaciguat, NO-Cbi, or low-dose estrogen replacement. Micro-CT analyses of tibiae showed that all three pharmacological interventions significantly improved trabecular bone architecture in ovariectomized animals, with similar effect sizes. Cinaciguat and NO-Cbi reversed ovariectomy-induced osteocyte apoptosis as efficiently as estradiol, and enhanced bone formation parameters in vivo, consistent with in vitro effects on osteoblast proliferation, differentiation, and survival. As previously reported, ovariectomy dramatically increased osteoclast numbers, and this effect was completely reversed by estradiol. NO-Cbi significantly decreased the number of osteoclasts in ovariectomized mice, whereas cinaciguat had only modest effects, suggesting cGMP-independent effects of NO-Cbi in osteoclasts.

## Conclusion

We conclude that estrogen deficiency represents a state of relative NO and cGMP deficiency, and that NO-dependent or NO-independent guanylate cyclase stimulation may represent a novel, anabolic treatment strategy for post-menopausal osteoporosis. These data confirm an important role of NO/cGMP signaling in bone biology.
